# Fungal urinary tract infection among chronic liver disease patients with hepatic encephalopathy and its treatment outcomes

**DOI:** 10.1002/jgh3.12470

**Published:** 2020-12-08

**Authors:** Lubna Kamani, Hamid Kalwar

**Affiliations:** ^1^ Department of Gastroenterology Liaquat National Hospital Karachi Pakistan

**Keywords:** Candida, chronic liver disease, fluconazole, fungal urinary tract infections, hepatic encephalopathy

## Abstract

**Background and Aim:**

Patients with chronic liver disease (CLD) are at high risk of infections, including fungal pathogens, which can lead to hepatic encephalopathy (HE) and increased mortality. Our aim is to evaluate the frequency and outcome of fungal urinary tract infections (FUTIs) in hospitalized patients with CLD and HE.

**Methods:**

This was a descriptive case series study using the nonprobability consecutive sampling technique, conducted at the Department of Gastroenterology, Liaquat National Hospital, Karachi, Pakistan. All patients above 18 years of age who were admitted with HE and CLD were enrolled after obtaining informed consent. Baseline laboratory investigation, urine detail report (UDR), and culture were sent on the day of admission. Fluconazole was started if the UDR reported yeast positivity. Data were analyzed using SPSS version 25.

**Results:**

A total of 236 patients were enrolled in this study. Mean age was 53.42 ± 5.567 years, and 95 (40.3%) were male. Urinary symptoms were present in 72 (30.5%) patients. Yeast positivity on UDR was present in 156 (66.1%), and 141 of 156 (90.3%) patients had urine culture positivity for fungal pathogen. A total of 55 patients died—36 (65.5%) in the FUTI group and 19 (34.5%) in the nonfungal UTI (NFUTI) group (*P* = 0.908). *Candida albicans* was the most common organism, present in 70 of 141 (49.6%) of patients. Predictors of mortality were renal insufficiency, hyperkalemia, hyponatremia, leukopenia, and advanced cirrhosis.

**Conclusion:**

FUTI in CLD patients with HE is common in hospitalized patients even without symptoms, and a high index of suspicion is required. *Candida albicans* was the most common organism. Prompt recognition and treatment can improve overall outcome.

## Graphical abstract

This study explains the frequency of fungal urinary tract infections in chronic liver disease patients with hepatic encephalopathy. A high index of suspicion is required for recognition and treatment of this infection, which can lead to improvement in outcome.

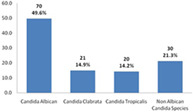

## Introduction

Chronic liver disease (CLD) patients are at high risk of developing infections that lead to life‐threatening conditions, such as sepsis and hepatic encephalopathy (HE).^1^ Such patients are at risk of infections for multiple reasons, including a dysfunctional immune response; increased permeability of the intestines, which causes alterations in the quantity and quality of the gut microbiota; and genetic predisposition, which contributes to the pathological translocation of fungal organisms from the gut to systemic circulation.^2^

The onset of fungal infections has been linked to the occurrence of multiple complications, for example, acute kidney injury (AKI), HE, and multiorgan failure, all of which confers long‐ and short‐term mortality.^3^ Fungal infections lead to a rise in the risk of mortality at any liver disease stage, either compensated or decompensated, and include acute or chronic liver failure as well.^4^ Among CLD patients, fungal infections are a common entity, with the Candida species being the most common. Fernandez et al. found that 2% of the patients having acute‐on‐chronic liver disease (ACLF) admitted in hospital had fungal infections. The presence of Candida species is reported in studies to be due to the failure of treatment.^5^

Candiduria is a commonly observed nosocomial infection of the urinary tract. Community‐acquired Candiduria is a rare entity in normal urinary tract anatomy, that is, in healthy population. The prevalence of fungal urinary tract infection (FUTI) is reported at about 10–15%, with the most isolated organism being Candida species.^6^

Candidemia can arise either endogenously or exogenously among liver disease patients; however, it is almost always caused due to prolongation in exposure to antibiotics as documented in some studies.^7^ An increase in the incidences of FUTI is linked to the usage of urinary catheters and treatment with broad‐spectrum antibiotics, immunosuppressive agents, and corticosteroids. Some other risks for FUTI include advanced age, predisposition to females, diabetic patients, chronic renal failure patients, and patients on hemodialysis.^8^ Early administration of antifungal treatments has been linked to improved outcomes, specifically among patients having severe infections.^9^ Among CLD patients, the efficacy and safety of antifungals is questionable as they appear to be associated with resistance to antifungals and demonstrate a decrease in the patient's tolerance and alterations in drug pharmacokinetics due to advanced liver disease.^10^ Even though fluconazole is still the most commonly used antifungal due to its liver‐suitable pharmacokinetics and tolerability, shifting toward non‐albicans strains have reported lower fluconazole susceptibility.^11^ The objectives of this study were to evaluate the frequency of FUTI in CLD patients with HE and also determine the outcome of its treatment.

## Methods

### *Study design*

This is a descriptive case series study using the nonprobability consecutive sampling technique, conducted at the Department of Gastroenterology, Liaquat National Hospital, Karachi, Pakistan. The primary aim of this study is to assess the frequency of FUTI in CLD with HE and the outcome of its treatment in terms of mortality.

### *Study population*

The study was conducted after approval by the hospital ethics committee and was conducted in accordance with Good Clinical Practice from December 2019 till May 2020, for a total of 6 months. Informed consent was obtained from all patients. The confidentiality of the data was maintained. A total of 236 patients of either gender aged older than 18 years with HE secondary to CLD due to viral cirrhosis and admitted in hospital were enrolled. The exclusion criteria were followed strictly to avoid confounding variables. A detailed history, including urinary symptoms, was obtained from the patient's next of kin. Patients with diabetes; with a history of use of steroids, immunosuppressants, antibiotics, or antifungal medications in the past 2 weeks; and having concomitant bacterial sepsis were excluded from the study. Other causes of HE, such as hematemesis, bacterial infections, use of sedatives, and hepatocellular carcinoma, were also excluded. Child‐Turcotte‐Pugh classification was used for classifying the status of CLD, while West Haven Grades of classification were used for classifying HE, and it was graded by a gastroenterology fellow or consultant.

### *Description of study assessments*

In 236 patients, the urine detail report (UDR) and culture and sensitivity (C/S) were carried out, with samples collected by sterile technique. It was set on the cysteine lactose electrolyte‐deficient agar plate, and suspected yeast colonies were isolated on Sabourauds dextrose agar with chromogenic medium and germ tube test, then processed by semiquantitative method using standard protocols was recorded. In catheterized patients, a urine sample was collected after sampling and cleaning the lower two‐thirds of Foley's catheter with pyodine and letting it dry. A urine sample was collected by the staff nurse and sent to the laboratory, where it was checked for the presence of pus cells, red blood cells, casts, crystals, or fungal organisms on direct microscopy. Blood culture, UDR, and C/S were sent on the day of admission, and treatment with fluconazole was started within 24 h of admission once the UDR report showed the presence of yeast. Fluconazole 100 mg I/V daily was started, and this was then switched to oral fluconazole once the patient was stable and demonstrated no encephalopathy for a total of 14 days. Later, fluconazole was changed according to the C/S of urine if needed. Treatment outcomes in terms of mortality were observed. All demography and clinical history was recorded. Ascitic fluid D/R and C/S were sent for patients with ascites. Patients were discharged once they were stable and fulfilled the discharge criteria of the hospital.

### *Statistical analysis*

Data were analyzed by using the Statistical Package for Social Sciences (SPSS, IBM SPSS Statistics) version 25. Mean and standard deviation were computed for quantitative variables, and frequency and percentage were calculated for qualitative variables. The chi square test was used to check the association between categorical variables. Odds ratios were calculated by univariate and multivariate binary logistic regression, and values ≤0.05 were considered significant.

## Results

**Figure 1:**
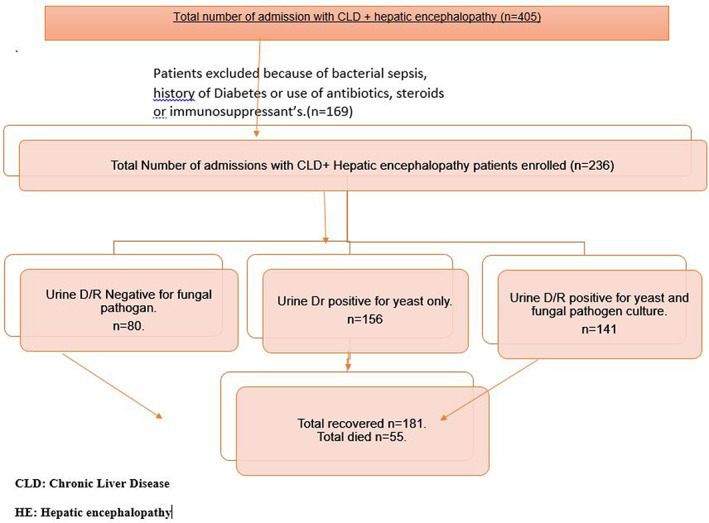
Chronic liver disease patients with hepatic encephalopathy.

In this study, a total of 236 patients were enrolled with CLD and HE (Fig. 1), and 95 patients (40.2%) were males. The mean age of the participants was 53.4 ± 5.5 years. Of 156 cases who were positive for yeast on UDR, 141 (90.3%) had urine culture positive for a fungal pathogen; 36 (15.2%) patients were classified as Child‐Pugh Class B, while 200 of 236 (84.7%) patients were in class C. Urinary symptoms at the time of admission were present in 72 (30.5%) patients (Table 1).

**Table 1:** Baseline demographics of the study participants

	*n* = 236 (%)
Gender	
Male	95 (40.3)
Female	141 (59.7)
Age (years)	
Mean ± SD	53.4 ± 5.5
Groups	
≤50 years	60 (25.4)
>50 years	176 (74.6)
TLC	
Mean ± SD	11 173.7 ± 7083.5
Groups	
≤5000	209 (88.6)
>5000	27 (11.4)
BMI (kg/m^2^)	
Mean ± SD	27.3 ± 1.8
Hospital stay (days)	
Mean ± SD	6.3 ± 2.2
Creatinine (mg/dL)	
Mean ± SD	1.6 ± 1.4
Groups	
≤1.2 mg/dL	108 (54.2)
>1.2 mg/dL	128 (54.2)
Potassium	
Mean ± SD	4.2 ± 0.9
Groups	
≤5	174 (73.7)
>5	62 (26.3)
Sodium (mg/dL)	
Mean ± SD	127.9 ± 5.3
Groups	
≤126 mg/dL	81 (34.3)
>126 mg/dL	155 (65.7)
Albumin (mg/dL)	
Mean ± SD	2.7 ± 0.4
Groups	
<2.7 mg/dL	65 (27.5)
≥2.7 mg/dL	171 (72.5)
Yeast positive on UDR	
Yes	156 (66.1)
No	80 (33.9)
C/S positivity (fungal pathogen) (*n* = 156)	
Yes	141 (90.3)
No	15 (9.6)
Type of fungus (*n* = 141)	
*Candida albicans*	70 (49.6)
Non‐Albicans Candida Species	30 (21.3)
*Candida tropicalis*	20 (14.2)
Candida Clabrata	21 (14.9)
Child class	
B	36 (15.3)
C	200 (84.7)
Outcome	
Recovered	181 (76.7)
Death	55 (23.3)

BMI, body mass index; SD, standard deviation; TLC, total leucocyte count; UDR, urine detail report.

The total number of patients who died was 55 (23.3%). In subgroup analysis, 36 (65.5%) patients died in the Fungal UTI (FUTI) group, and 19 (34.5%) died in the Nonfungal UTI (NUTI) group. There was an insignificant association of mortality with FUTI (*P* = 0.908). Of 156 patients with yeast‐positive urine D/R, 141 (90.3%) patients had culture positivity for a fungal pathogen. However, with respect to the type of fungus, no significant association was noted in the mortality (*P* = 0.066) (Table 2).

**Table 2:** Outcomes in type of fungal organism isolated on urine

Variable	Recovered (*n* = 181), *n* (%)	Death (*n* = 55), *n* (%)	*P*‐value
Yeast positive on UDR			
Yes	120 (66.3)	36 (65.5)	0.51
No	61 (33.7)	19 (34.5)	
C/S positivity (fungal pathogen)			
Yes	114 (63)	27 (49.1)	0.04
No	67 (37)	28 (50.9)	
Type of fungus			
Candida Albicans	56 (30.9)	14 (25.5)	0.298
Non‐Albicans Candida Species	24 (13.3)	6 (10.9)	
Candida Tropicalis	15 (8.3)	5 (9.1)	
Candida Clabrata	19 (10.5)	2 (3.6)	

UDR, urine detail report.

**Figure 2:**
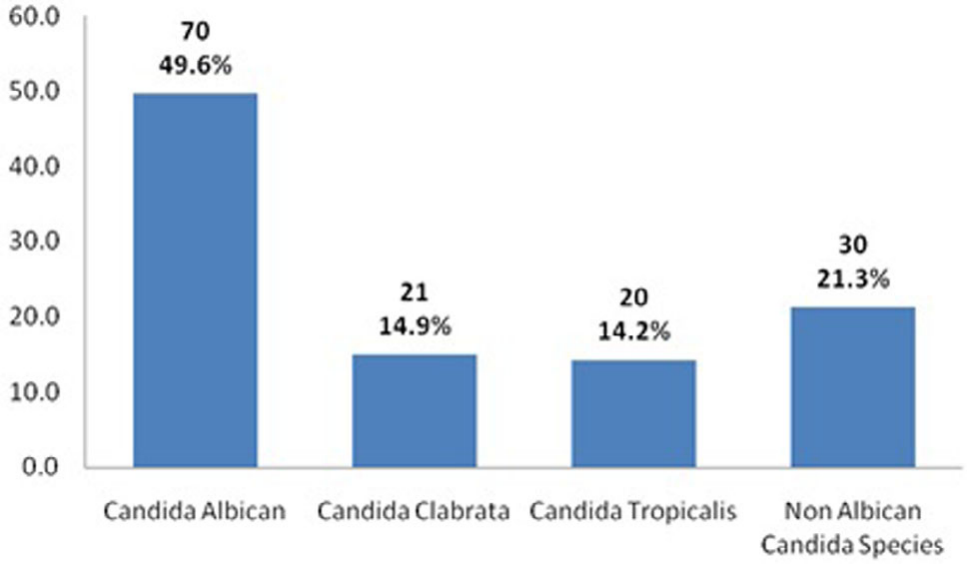
Graphical representation of the type of fungal organism isolated on urine culture (n=141).

*Candida albicans* species was most the common fungal pathogen in urine culture and was observed in 70 of 141 (49.6%) patients, followed by non‐albicans candida species in 30 (21.3%) patients (Fig. 2). In univariate analysis renal insufficiency, hyperkalemia, hyponatremia, and leukopenia were predictors of mortality. Patients in Child‐Pugh Class C have a high chance of mortality (odds ratio [OR] = 1.308). (Table 3). In multivariate analysis, renal insufficiency, hyperkalemia, leukopenia, hyponatremia, and Child‐Pugh Class C were predictors of mortality (Table 4). The presence of a fungal pathogen in urine does not increase the risk of mortality both in univariate and multivariate analyses. None of the patients had fungal pathogen positivity in ascetic fluid culture or blood culture.

**Table 3:** Univariate analysis for predictors of mortality

	Univariate analysis			
	*P*‐value	Odds ratio	(95% CI)	
Gender				
Female		1		
Male	0.965	0.986	0.533	1.826
Age group				
≤50 years		1		
>50 years	0.288	0.697	0.358	1.356
TLC count				
≤5000®		1		
>5000	<0.001	0.108	0.045	0.258
Creatinine				
≤1.2 mg/dL		1		
>1.2 mg/dL	<0.001	17.221	5.969	49.680
Potassium				
≤5®		1		
>5	<0.001	3.243	1.707	6.163
Child class				
Class B		1		
Class C	0.553	1.308	0.539	3.176
UTI				
Nonfungal UTI		1		
Fungal UTI	0.908	0.963	0.510	1.818
Sodium				
>126 mg/dL		1		
≤126 mg/dL	<0.001	48.387	17.848	131.178

CI, confidence interval; TLC, total leukocyte Count; UTI, urinary tract infection.

**Table 4:** Multivariate analysis for predictors of mortality

	Multivariate analysis			
	*P*‐value	Odds ratio	(95% CI)	
TLC count				
≤5000		1		
>5000	<0.001	0.080	0.015	0.428
Creatinine				
≤1.2 mg/dL		1		
>1.2 mg/dL	<0.001	16.915	3.803	75.236
Potassium				
≤5®		1		
>5	<0.001	10.604	2.913	38.603
Child class				
Class B		1		
Class C	<0.051	4.294	0.993	18.561
UTI				
Nonfungal UTI		1		
Fungal UTI	0.06	0.344	0.113	1.049
Sodium				
>126 mg/dL		1		
≤126 mg/dL	<0.001	68.827	17.148	276.252

CI, confidence interval; TLC, total leucocyte count; UTI, urinary tract infection.

## Discussion

Studies have reported a significant rise in the incidence of fungal infections in CLD patients, the main reason being ineffective immune mediation and unrestricted use of antibiotics. Funguria is specifically a devastating condition as no standard criteria exist that could ascertain if a positive culture is significant and if treatment is a necessity and, when indicated, which treatment will be the most effective. As much information is not available regarding funguria, this topic still remains a matter of debate.^12^, ^13^

In our study, approximately more than two‐thirds of patients admitted with CLD and HE had yeast positivity on UDR, although only less than a third had urinary symptoms. Early recognition of a fungal pathogen is required in CLD patients as it is a treatable cause of HE. FUTI was more common in women, and the likely explanation is the anatomy of their urinary tract, which has a short urethra.

In line with our study, Storfer *et al*., reported, using 105 patients in whom *C. albicans* had the highest frequency of fungal organism isolates, recorded in 56 (53%) patients, that FUTI was also more prevalent among women.^14^

Advanced hepatic disease is considered a risk factor for fungal infections, mainly because of high gastrointestinal permeability leading to favorable translocation of large pathogens like fungus, and these patients are malnourished with severe immune dysfunction.^15^ Furthermore, in our study, compared to Child‐Turcotte‐Pugh (CTP) class B, the ORs of mortality in CTP class C on univariate and multivariate analyses were (OR = 1.308) and (OR = 4.294), respectively. Other reported risk factors for fungal infections include prolonged hospitalizations and invasive procedures.^16^ Research has observed a high mortality rate among patients with fungal infection having liver or kidney disease.^17^ A retrospective study of 120 liver disease patients reported fungal colonization as an independent risk factor of mortality (*P* = 0.047).^18^ On the contrary, in our study, the presence of FUTI was not among the predictors of mortality, and a possible explanation is that patients in our study with UDR positivity for yeast were started on antifungal medication within 24 h of admission. The treating doctor must have a high index of suspicion for the early identification of fungal infection among patients with cirrhosis of the liver.^19^

Another study of 185 liver disease patients having positive cultures for the Candida species found that only 47% of the patients with fungal infections were treated with antifungal drugs, while the remaining patients died.^20^ Likewise, in a study with 126 patients having positive fungal infection cultures, only 43% of patients received antifungal medication. Candida infection has been linked with prolonged hospitalizations, prior surgeries, central line placement, neutropenia, and prior use of antibiotics.^21^ Bassetti et al. reported that, in 169 patients with fungal infection, 46% of patients were infected with non‐candida species, and 35% of mortality was linked to candida infection and absent or inadequate antifungal treatment.^22^ Rasool et al.^23^ reported renal insufficiency as one of the predictors of mortality in fungal infections in CLD patients, and similar findings were observed in our study.

In this study, 36 of 55 patients (65.45%) who died had FUTI, and the results were similar to those of a study conducted by Lahmer T et al. on cirrhotic patients with or without fungal colonization, where the reported mortality was 78 *versus* 35%; *P* < 0.001.^18^ Fungal infections frequently occur, in around 40–45% of patients, when antifungal prophylaxis is not started or completed, especially in liver disease patients.^24^ The most common cause of fungal infections has been the liver status, patient baseline characteristics, geographical fungal ecology, and resistance.^25^ Thomas et al. reported a decrease in mortality rate to 40% in patients treated with fluconazole for fungal infection s compared to 80% in untreated cases.^26^Our study has some limitations, such as being a single‐center, observational study with a limited sample size, and a repeat urine culture was also not performed after the completion of antifungal treatment to document fungal clearance.

Ignoring funguria in advanced CLD patients can lead to invasive candidiasis and septicemia with devastating outcomes. An aggressive approach by the treating physician is warranted, which can lead to better outcomes.

## Conclusion

FUTI in CLD patients with HE is common in hospitalized patients, and a high index of suspicion is required, with *Candida albicans* being the most common organism. Prompt recognition and treatment can improve overall outcome.
